# Effect of Propofol versus Sevoflurane Anesthesia on Acute Kidney Injury after Lung Transplantation Surgery: A Prospective Randomized Controlled Trial

**DOI:** 10.3390/jcm11226862

**Published:** 2022-11-21

**Authors:** Young Song, Hyo-Chae Paik, Namo Kim, Heejae Jung, Jin-Gu Lee, Young-chul Yoo

**Affiliations:** 1 Department of Anesthesiology and Pain Medicine, Anesthesia and Pain Research Institute, Yonsei University College of Medicine, Seoul 03722, Republic of Korea; 2 Thoracic and Cardiovascular Surgery, Yonsei University College of Medicine, Seoul 03722, Republic of Korea; 3 Department of Anesthesiology and Pain Medicine, Yonsei University College of Medicine, Seoul 03722, Republic of Korea

**Keywords:** acute kidney injury, lung transplantation, inflammation, anesthetics, propofol, sevoflurane

## Abstract

This prospective randomized controlled trial aimed to compare the effects of sevoflurane and propofol anesthesia on the occurrence of acute kidney injury (AKI) following lung transplantation (LTx) surgery. Sixty adult patients undergoing bilateral LTx were randomized to receive either inhalation of sevoflurane or continuous infusion of propofol for general anesthesia. The primary outcomes were AKI incidence according to the Acute Kidney Injury Network (AKIN) criteria and blood biomarker of kidney injury, including neutrophil gelatinase-associated lipocalin (NGAL) and cystatin C levels within 48 h of surgery. Serum interleukin (IL)-1β, IL-6, tumor necrosis factor-α, and superoxide dismutase were measured before and after surgery. The post-operative 30-day morbidity and long-term mortality were also assessed. Significantly fewer patients in the propofol group developed AKI compared with the sevoflurane group (13% vs. 38%, *p* = 0.030). NGAL levels were significantly lower in the propofol group at immediately after, 24 h, and 48 h post-operation. IL-6 levels were significantly lower in the propofol group immediately after surgery. AKI occurrence was significantly associated with a lower 5-year survival rate. Total intravenous anesthesia with propofol reduced the AKI incidence in LTx compared with sevoflurane, which is understood to be mediated by the attenuation of inflammatory responses.

## 1. Introduction

Acute kidney injury (AKI) is the most frequent complication following lung transplantation (LTx), occurring in up to 70% of recipients [1,2,3]. Perioperative renal damage is closely associated with serious morbidity, including permanent renal failure and death after LTx [1,3,4,5]. Unfortunately, most of the precipitating factors, including lung ischemia–reperfusion (IR), extracorporeal circulation during the procedure, surgical stress, multiple transfusion, and use of nephrotoxic agents [3,6], are not modifiable; moreover, no preventive strategies against AKI in LTx patients have been established.

The organ-protective effects of inhalational anesthetics are well-established. Specifically, attenuation of IR injury in the vital organs by sevoflurane via pro-survival signaling pathways, or the so-called pharmacologic conditioning effect, has been consistently demonstrated [7]. Meanwhile, the most widely used intravenous anesthetic, propofol, has also exhibited organ-protective properties against IR for decades [8]. Both agents have also consistently been reported to reduce the inflammatory response and oxidative stress elicited by surgery or critical illnesses, such as sepsis, thereby attenuating organ damage [9,10,11]. Since the kidneys are highly vulnerable to IR, inflammation, and oxidative stress [12,13], renoprotection by general anesthetic methods during high-risk surgery has received great attention in particular [9]. Naturally there have been robust investigations into the comparison of inhalational anesthesia with sevoflurane and total intravenous anesthesia (TIVA) with propofol in surgeries that are accompanied by IR injury to the kidneys, systemic inflammation, and oxidative stress, which include cardiac surgery, organ transplant, and laparoscopic abdominal surgery [14,15,16,17]; however, the overall results are controversial. 

Both anesthetic agents may confer renoprotection during LTx, yet little is known about this application. Hence, we conducted a prospective randomized controlled trial to assess which anesthetic methods are superior in reducing the incidence of AKI in patients undergoing LTx. For early and sensitive detection, plasma biomarkers for AKI were assessed in addition to serum creatinine and urine output. We also evaluated the blood levels of pro-inflammatory cytokines and a marker of oxidative stress to provide insights into the underlying mechanisms at play. 

## 2. Materials and Methods

### 2.1. Ethics and Patient Enrollment

The study was conducted at the Yonsei University Health System, Seoul, Republic of Korea, between January 2014 and August 2016 after approval by the Institutional Review Board of Severance Hospital at Yonsei University College of Medicine (IRB# 4-2013-0648, date of approval: 8 November 2013) in accordance with the Helsinki Declaration, 2013. The trial was registered prior to patient enrollment at clinicaltrials.gov (NCT02009280, date of registration: 11 December 2013). Written informed consent was obtained from all participants. Patients aged ≥ 20 years scheduled for isolated double LTx with extracorporeal membrane oxygenation (ECMO) support were considered eligible. Exclusion criteria were body mass index > 30 kg/m^2^ to avoid overdosing; known allergies to propofol, egg protein, or soybean emulsion; recent self-administration of antioxidants, such as vitamin C; donor age ≥ 70; and moderate to severe chronic kidney disease that was defined as estimated glomerular filtration rate (eGFR) < 60 mL/min/1.73 m^2^—as well as refusal to participate in the study. 

### 2.2. Study Protocol

Patients were randomly allocated into either the sevoflurane or propofol groups immediately after enrollment using R Statistical Software (Foundation for Statistical Computing, Vienna, Austria) and sealed in opaque, sequentially numbered envelopes. In the sevoflurane group, anesthesia was induced with midazolam (50 µg/kg), sufentanil (1.0–3.0 µg/kg), and rocuronium bromide (0.9 mg/kg), and was maintained with sevoflurane at an end-tidal concentration of 0.4–1.5% (within age-corrected 0.5–1.5 minimum alveolar concentration) and continuous infusion of sufentanil (0.3–0.5 µg/kg/h) to achieve a bispectral index score between 40–60. In the propofol group, anesthesia was induced with propofol (1 mg/kg), sufentanil (1.0–3.0 µg/kg), and rocuronium bromide (0.9 mg/kg), and maintained with continuous infusion of propofol (60–250 µg/kg/min) [14] and sufentanil (0.3–0.5 µg/kg/h) to achieve a bispectral index score between 40–60. Sevoflurane or propofol anesthesia was performed by anesthesiologists who were responsible for the intra-operative management and not involved in the current study. The participants, surgeons, intensive care unit (ICU) medical team, and anesthesiologists responsible for data collection were blinded until the end of the study. 

### 2.3. Perioperative Care

According to institutional standards [18], veno-arterial (VA) ECMO was instituted after induction of anesthesia. The surgeries were sequentially performed under clamshell incision, starting from the right-side surgery. Lungs, which had been harvested en bloc from brain-dead donors and stored in a low-potassium dextran solution (Perfadex^®^; Duraent Biologicals, Hyderabad, India), were implanted with the bronchial and vascular anastomoses. The VA ECMO using the Bioline heparin-coated Quadrox PLS circuit system (Maquet Cardiopulmonary, Hirrlingen, Germany) primed with 0.8 L of acetated Ringer’s solution (Plasma Solution A Inj.; CJ Pharma, Seoul, Republic of Korea) was performed with femoro artery–femoral vein cannulation. A 15–17 Fr sized arterial cannula for arterial inflow was inserted into the common femoral artery, followed by 20–24 Fr sized cannula insertion into common femoral vein. Before initiating ECMO circulation, unfractionated heparin was injected with targeting for an activated clotting time between 130 and 150 s. During surgery, total ECMO flow was maintained at approximately 60 mL/kg/min. Crystalloid solution was infused at a rate of 6–8 mg/kg/h and colloid solution (Volulyte; Fresenius Kabi, Bad Homburg, Germany) was administered up to 20 mL/kg per day to compensate for the blood loss. Mean arterial pressure was maintained between 60 and 80 mmHg during the operation with the use of norepinephrine and vasopressin. Milrinone was administered in patients with pre-existing and/or newly developed right ventricle failure or moderate to severe pulmonary hypertension. Protective ventilation was commenced after right side reperfusion and ECMO were weaned off after left-side reperfusion in patients with a ratio of arterial O_2_ partial pressure (PaO_2_)/fractional inspired O_2_ (FiO_2_) > 100, cardiac index (CI) ≥ 2.0 L/min/m^2^, and MAP > 60 mmHg. In those patients that achieved targeted CI and MAP, but with PaO_2_/FiO_2_ < 100, the VA ECMO was converted to veno-venous ECMO before the patient left the operating room (OR). VA ECMO support was continued in patients who did not achieve any of the goals. Packed red blood cells and blood salvaged by a cell saver used when the patient’s bronchus was closed were transfused when hematocrit was <27%. Transfusion of fresh frozen plasma and platelets was in accordance with the institutional guidelines [19]. Intravenous furosemide was repeatedly administered with an escalating dose of 10–80 mg once urine output decreased below 1 mL/kg/h for 30 min. When fluid overload, oliguria, or hyperkalemia persisted or metabolic acidosis (pH < 7.1) was observed in spite of maintenance of normal renal perfusion pressure and furosemide challenges, renal replacement therapy was started. Immunosuppressive therapy consisting of a calcineurin inhibitor (Tacrolimus; FK506), mycophenolate mofetil, and low-dose prednisolone was started from day 1 post-operation. 

### 2.4. Study Endpoints

The primary endpoint was the incidence of post-operative 48 h AKI according to the Acute Kidney Injury Network (AKIN) criteria, which is as follows [20]: stage I = an absolute increase in serum creatinine ≥0.3 mg/dL, an increase to 150–200% of baseline value, or urine output < 0.5 mL/kg/h for ≥6 h; stage II = serum creatinine increase to 200–300% of baseline or urine output <0.5 mL/kg/h for ≥12 h; stage III = serum creatinine ≥4 mg/dL (with an acute increase of ≥0.5 mg/dL) or increase to >300% of baseline, urine output <0.3 mL/kg/h for ≥24 h or anuria for 12 h, or need for renal replacement therapy. Serum creatinine levels were assessed immediately before and at the end of surgery, as well as at least twice per 24 h. Blood levels of biomarkers for AKI, including neutrophil gelatinase-associated lipocaline (NGAL) and Cystatin C, were assessed immediately before and after surgery, at 24 h, and at 48 h after surgery. Blood serum levels of IL-1β, IL-6, tumor necrosis factor (TNF)-α, and superoxide dismutase (SOD) were assessed using the Human IL-1β Quantikine ELISA kit, IL-6 Quantikine ELISA kit, and TNF-α Quantikine HS ELISA kits (R&D System Inc., Minneapolis, MN, USA) and Human SOD ELISA Kit (MyBioSource, San Diego, CA, USA), respectively, in 19 patients from each group immediately before and after surgery.

### 2.5. Other Assessments

Pre-operative patient characteristics, including age, sex, body mass index, history of hypertension and diabetes, left ventricular ejection fraction, renal function determined by eGFR, primary diagnosis, waitlist time, forced vital capacity and forced vital capacity in 1 s measured by spirometry, mean pulmonary arterial pressure, and ventilatory and ECMO support were recorded. Perioperative data collection included donor age and sex, ischemic time, surgery time, prolonged hypoxia (SpO_2_ < 90% over 15 min), fluid intake, blood transfusion, urine output, and blood loss during operation and 48 h post-operation, ECMO wean-off or not before leaving the OR, milrinone and vasopressor use during surgery and 48 h post-operation, and as a post-operative requirement of RRT within 48 h and after 1 month. Post-operative 1-month morbidity endpoints included re-operation for bleeding, acute rejection [21], newly developed cerebrovascular accident, grade 3 primary graft dysfunction [22], duration of ECMO and ventilatory support after surgery, re-insertion of ECMO after surgery, length of ICU and hospital stay, and mortality. Five-year mortality was also assessed as an indicator of long-term prognosis.

### 2.6. Statistical Analysis

Sample size calculation was performed using PASS version 11.0.7 (NCSS Statistical Software, 2013). Incidence of renal complication following LTx in our institution was 50% according to our medical record database, which is similar to the report from a recent meta-analysis [23]. Expecting a reduction of 70% in the incidence of AKI with propofol anesthesia versus sevoflurane based on our previous study of cardiac surgery [14], 27 were required in each group in order to obtain a power of 80% when considering a type I error of 0.05. Assuming a 10% dropout rate, we decided to enroll a total of 60 patients.

Results are shown as mean ± SD, median [interquartile range], or number of patients (%). After the Shapiro–Wilk test for normality, continuous variables between the groups were compared using the independent *t*-test or Mann–Whitney U test. Repeated measure variables were analyzed using a linear mixed model with patient indicator as a random effect, and group, time, and group-by-time as fixed effects. When the interactions of group, time, and group-by-time of the variables showed statistical significance, a post-hoc analysis was carried out with Bonferroni correction for the adjustment for multiple comparisons. Categorical variables between the groups were compared using the chi-Square or Fisher’s exact test when appropriate. Survival curves were constructed to compare long-term survival rates between the groups using the Kaplan–Meier method and log-rank test. Statistical analyses were performed using SPSS version 23 (IBM Corp., Armonk, NY, USA) and SAS version 9.4 (SAS Institute, Cary, NC, USA) and a *p*-value < 0.05 was considered statistically significant.

## 3. Results

### 3.1. Patient Characteristics

**Figure 1:**
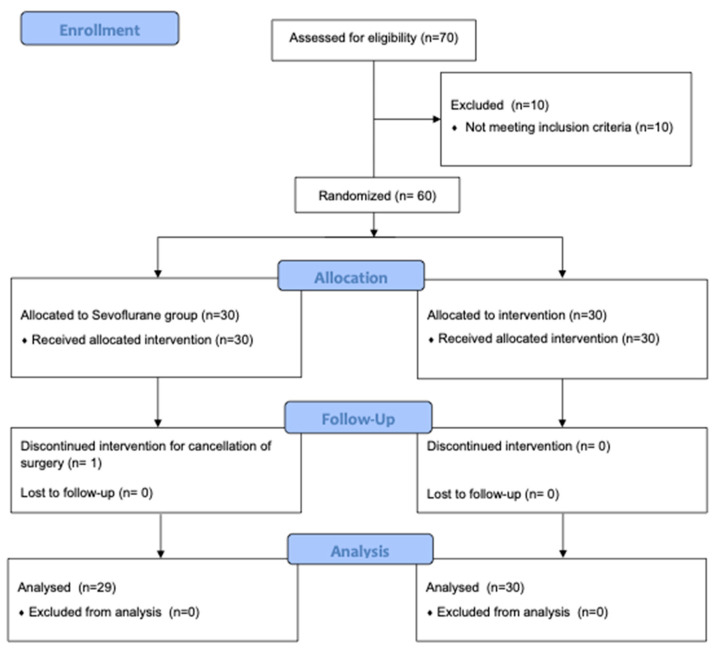
CONSORT diagram showing the flow of participants through each stage of the randomized trial.

Seventy patients were assessed for eligibility and 10 patients were excluded before randomization. Among the 60 enrolled patients, 1 in the sevoflurane group was excluded from the analysis due to cancellation of surgery. Thus, a total of 59 patients were included in the final analysis (sevoflurane group, 29; propofol group, 30) (Figure 1). All of them were transplanted with VA ECMO and none needed additional CPB support.

The patient characteristics are summarized in Table 1. The demographic factors, primary lung disease, and pre-operative conditions, including pulmonary function, mean PA pressure, need for cardiorespiratory support, and renal function, were comparable between the groups.

### 3.2. Perioperative Data

Donor age and sex, graft ischemic time, surgery time, fluid intake, transfused blood volume, urine output, blood loss, amount of milrinone and vasopressors administered during surgery and 48 h post-operation, proportion of patients who experienced prolonged hypoxia during surgery, and patients who were weaned off ECMO at the OR were not different between the groups (Table 2).

### 3.3. Perioperative Renal Function and Post-Operative 30-Day Morbidity

As shown on Table 3, the baseline blood creatinine was not different between the groups. Post-operative AKI according to the AKIN criteria occurred in 11 patients (38%) of the sevoflurane group compared with 4 patients (13%) of the propofol group (*p* = 0.030). The proportion of stage I, II, and III AKI patients was not significantly different between the groups, but no one in the propofol group developed stage II or III AKI, compared with four patients in the sevoflurane group. The interaction of group and time in blood levels of NGAL was significant between the groups in the linear mixed model analysis (*p* = 0.039). The post-hoc Bonferroni correction revealed that the levels were significantly lower in the propofol group at immediately after, 24 h, and 48 h after surgery, compared with the sevoflurane group. The blood levels of cystatin C did not differ between the groups. Furthermore, the dosage of administered furosemide was not different between the groups. The RRT requirement within 48 h post-operation was not significantly different between the groups (*p* = 0.052). Post-operative 1-month morbidity, mortality, and length of stays at ICU and hospital were comparable between the groups (Table 3). The incidence of grade 3 PGD was also not significantly different between the groups (27% and 48% in propofol group and sevoflurane group, respectively, *p* = 0.086). The post-operative mortality at 1, 3, and 5 years after surgery were not different between the groups.

**Figure 2:**
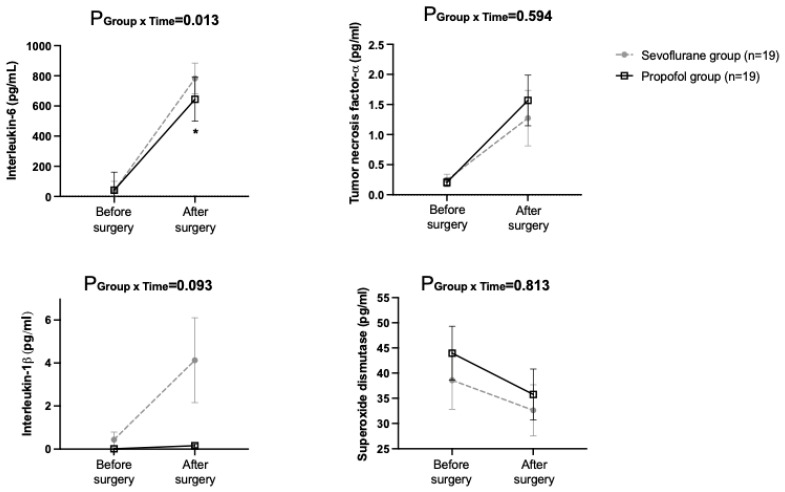
Serum levels of inflammatory cytokines and antioxidant enzymes. P_GroupxTime_ = *p*-value of the group and time interaction obtained by the linear mixed model.

The interaction of group and time in serum IL-6 levels was significant between the groups (*p* = 0.013). The post-hoc Bonferroni correction revealed that the level were significantly lower in the propofol group at immediately after surgery, compared with the sevoflurane group. There were no differences in the IL-1β, TNF-α, or SOD levels between the groups (Figure 2).

### 3.4. Long-Term Prognosis of AKI

**Figure 3:**
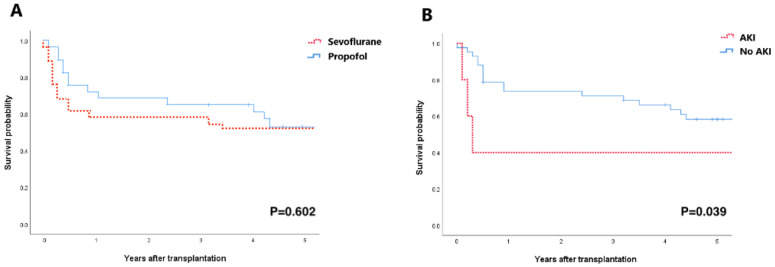
Kaplan–Meier analysis of the 5-year survival rate after surgery. (**A**) Survival curves according to the anesthesia agents. (**B**) Survival curves according to the post-operative AKI development. AKI = acute kidney injury.

According to Kaplan–Meier estimates, the 5-year survival rate did not differ between the propofol group and the sevoflurane group (Figure 3A); however, patients who developed post-operative AKI had a significantly lower 5-year survival, compared to those that did not develop AKI (log-rank *p* = 0.039, Figure 3B).

## 4. Discussion

We demonstrate in the present study, which is the first randomized clinical trial addressing LTx, that TIVA with propofol reduced the AKI incidence according to the AKIN criteria, compared with that attained with sevoflurane anesthesia. The blood NGAL levels, an effective biomarker of acute tubular injury, was also less increased until 48 h post-surgery in the propofol group. The blood serum levels of IL-6 after surgery were significantly lower in the propofol group, indicating the stronger anti-inflammatory properties of propofol versus sevoflurane as the primary mechanism for our result. This superior renoprotection was not associated with a better survival rate; however, patients who developed AKI showed a significantly lower 5-year survival compared with those who did not, regardless of type of anesthetics.

Since both anesthetic agents are known to protect the kidneys, a comparison between the effects of sevoflurane anesthesia and TIVA with propofol on the post-operative renal outcomes have been fairly hotly debated for a long time [14,15,16,17]; however, only a few sophisticated clinical studies have assessed transplant surgery despite the high risk of perioperative renal insult, which show conflicting results. In a randomized clinical trial on pediatric liver transplantation [16], patients receiving sevoflurane anesthesia were less likely to develop AKI and exhibited lower levels of serum TNF-α and IL-18 from the reperfusion until 24 h after surgery, compared with those associated with patients receiving TIVA. On the other hand, our previous clinical trial on the adult living donor liver transplantation revealed no difference in the incidence of AKI between inhalational anesthesia and TIVA [17].

The main reason for such inconsistencies between the studies stem from the fact that the efficacies of anesthetic agents depend on the type of organ encountered IR and mechanisms of injury according to the type of surgery. In liver transplant surgery, the macrophage, the primary source of IL-18, is markedly activated by the inferior vena cava clamp during the an-hepatic phase. A previous in-vitro study reporting that macrophagic activity was inhibited by sevoflurane, but not by propofol, partly explains the lower incidence of AKI and serum levels of IL-18 [24,25] in the pediatric liver recipients with sevoflurane anesthesia [17]; moreover, because enterobacteria massively shifts toward systemic circulation and reaches the kidneys during liver transplant surgery [26], sevoflurane anesthesia could be more protective than TIVA because it is known to favorably affect the internal microbiome, thereby enhancing the integrity of the intestinal mucosal barrier [27]. Meanwhile, IR of the lungs may result in an explosive inflammatory reaction, since the lungs are particularly dependent on cytokine-regulated defense mechanisms throughout life as they continuously interact with the external environment [28]; moreover, because the lungs are harvested from a braindead donor in most cases, the greater amount of pro-inflammatory cytokines and chemokines, adhesion molecules, and stress hormones were likely to have been systematically released at reperfusion, which is in contrast to organs obtained from living donors [29,30]. Our immunoassay exhibiting lower blood IL-6 levels in the propofol group indicated that propofol’s stronger anti-inflammatory properties in comparison with sevoflurane may be a possible mechanism of renoprotection in LTx, although its upstream pathway was not clearly demonstrated here.

More importantly, propofol should have attenuated renal insults elicited by the extracorporeal circulation during LTx better than that attained via sevoflurane. We previously reported a similar result for an on-pump valvular heart surgery during which propofol anesthesia reduced post-operative AKI occurrence and blood levels of IL-6 and C-reactive protein, compared with those associated with sevoflurane anesthesia [14]. Despite the beneficial effects of ECMO over CPB support during LTx, ECMO itself poses a high risk of renal damage associated with pro-inflammatory cytokine activation induced by continuous exposure of the blood to the non-endothelialized and non-physiological ECMO interface [31,32]. In a recent clinical trial assessing the immune-modulating effects of TIVA versus sevoflurane anesthesia during cardiac surgery with CPB, TIVA resulted in higher blood levels of monocyte subsets with high CD163 surface expression and low human leukocyte antigen (HLA)-DR expression, which is known to produce various anti-inflammatory mediators and exert cytoprotective properties in situations of extracorporeal circulation [33]; moreover, the greater blunting effect of propofol on the sympathetic activity induced by extracorporeal circulation and surgical stress could have been attributed to the better renoprotection of propofol anesthesia, as the release of adrenalin and renin impairs renal blood flow and evokes IL-6 secretion [34,35].

Despite its renoprotective performance, propofol anesthesia did not improve the survival rates in our study. This could be attributable to several decisive factors for mortality, such as PGD, which is mainly determined by severity of pulmonary IRI and influenced by numerous donor and recipient factors [36]. We initially expected the occurrence of PGD to be further decreased via one of those anesthetic strategies; however, it was not significantly different between the groups, which might have been due to the relatively small sample size for the comparison considering the beneficial effects of both agents on the lung IRI [37,38]. Conversely, AKI was significantly related to a lower 5-year survival, which is consistent with previous reports on the relationship between the post-LTx AKI and short-and long-term mortality [5,6]. It would be feasible to further evaluate the effects of anesthetics on long-term mortality after LTx with a larger sample size.

There are several limitations that should be addressed in the current study. (1) Although the concentration of sevoflurane was sufficient to maintain the hypnotic state within the expected range, representing adequate general anesthesia for surgery, whether or not the blood concentration could have attained a level sufficient for exerting renoprotection is open to debate because of the loss of gas diffusion capacity in the lung recipients until the new lungs start functioning; however, we selected clinically relevant doses of both agents, which were not to be exceeded for safety reasons. (2) Many factors including the patients’ co-morbidities, duration of ECMO or ventilatory support pre- and post-surgery, duration and severity of hypoxia, and perioperative hemodynamic instability may influence the protective efficacies of anesthetic agents. Although these data were comparable between the groups, we cannot completely exclude the possibility of confounding effects of these variables as well as other factors that we might have missed. (3) Given the well-known antioxidative stress property of propofol [39], it is difficult to conclude that renoprotection in the current study was unrelated to propofol’s attenuation of oxidative stress. Some inaccuracy exists in measuring the degree of oxidative stress using body fluids in human [40]. Although the blood SOD level is an established indicator of anti-oxidant capacity, the possibility of false negative results cannot be excluded because of the lack of additional measures of free-radical production or lipid oxidation products. (4) We used midazolam for induction of anesthesia in the sevoflurane group, but not in the propofol group. The anti-inflammatory action of midazolam could have had an influence on cytokine levels [41,42], although the effect was assumed not to be remarkable.

## 5. Conclusions

In conclusion, TIVA with propofol was effective in reducing the AKI incidence in patients undergoing LTx compared with that associated with sevoflurane inhalational anesthesia. This may be related to propofol’s ability to attenuate the systemic inflammatory responses to lung IR, surgical trauma, and extracorporeal circulation during surgery; however, the beneficial effect was not related to long-term prognoses. Since it was a single-center study with a relatively small sample size, larger investigations are needed to confirm the current results.

## Figures and Tables

**Table 1:** Patient characteristics.

Variables	Sevoflurane Group (n = 29)	Propofol Group (n = 30)	*p* Value	
Age, y	51 ± 14	54 ± 11	0.381	
Male sex	17 (59)	16 (53)	0.908	
Body surface area, m^2^	1.7 ± 0.2	1.6 ± 0.1	0.095	
Hypertension	5 (17)	4 (13)	0.731	
Diabetes	5 (17)	5 (17)	1.000	
Left ventricular ejection fraction, %	61 ± 13	62 ± 14	0.761	
Estimated glomerular filtration rate, mL/min/1.73 m^2^	111.2 ± 22.9	110.3 ± 15.7	0.854	
Primary diagnosis leading to transplantation				
Idiopathic pulmonary fibrosis	12 (41)	16 (53)	0.358	
Bronchiectasis	5 (17)	2 (7)	0.254	
Interstitial lung disease	5 (17)	7 (23)	0.561	
GVHD after HSCT	5 (17)	1 (3)	0.103	
Chronic obstructive pulmonary disease	0	2 (7)	0.492	
Primary pulmonary hypertension	1 (3)	0	0.492	
Others	1 (3)	2 (7)	1.000	
Waitlist time, days	62 [6–141]	97 [34–146]	0.318	
FVC (% predicted)	43 [33–47]	38 [32–51]	0.335	
FEV1 (% predicted)	50 ± 23	43 ± 20	0.234	
Mean PA pressure, mmHg	27 ± 7	25 ± 8	0.446	
Ventilatory support	9 (31)	4 (13)	0.101	
ECMO support	6 (21)	3 (10)	0.299	

Data are expressed as mean ± SD, median [IQR], or number of patients. GVHD = graft-versus-host disease; HSCT = hematopoietic stem cell transplant; FVC = functional vital capacity; FEV1 = forced expiratory volume in 1 s; PA = pulmonary arterial; ECMO = extracorporeal membrane oxygenation.

**Table 2:** Perioperative data.

Variables	Sevoflurane Group (n = 29)	Propofol Group (n = 30)	*p* Value
Donor age	42 ± 12	41 ± 13	0.805
Sex matching, %	23 (79)	18 (60)	0.107
Ischemic time, h	244 ± 84	237 ± 84	0.750
Intraoperative data			
Surgery time, min	397 ± 54	372 ± 72	0.165
Fluid input, mL	10263 ± 3592	10402 ± 5408	0.549
pRBC transfusion, mL	1820 [1000–2700]	1500 [1200–2400]	0.853
FFP transfusion, mL	260 [0–390]	130 [0–390]	0.475
Platelet transfusion, mL	240 [0–240]	0 [0–240]	0.500
Urine output, mL	1225 [720–2130]	953 [800–2440]	0.848
Blood loss, mL	2050 [3500–14,000]	1925 [1100–2500]	0.473
Norepinephrine administered, (μg/kg/min)/n	0.03 [0.02–0.07]/27	0.04 [0.02–0.06]/30	0.788
Vasopressin administered, (IU)/n	3.0 [0.4–7.0]/14	4.0 [2.0–7.5]/15	0.664
Milrinone administered, (mg)/n	5.0 [0–9.5]/20	4.7 [2.4–7.2]/26	0.994
SpO_2_ below 90% > consecutive 15 min	3 (10)	2 (7)	>0.999
ECMO wean-off at the OR	13 (45)	20 (67)	0.091
Postoperative data for 48 h			
Fluid input, mL	7586 ± 1274	7125 ± 984	0.124
pRBC transfusion, mL	250 [0–500]	250 [0–250]	0.664
FFP transfusion, mL	0 [0–390]	260 [0–390]	0.857
Platelet transfusion, mL	240 [0–240]	240 [0–240]	1.000
Urine output, mL	5468 [4280–6822]	5987 [4922–6892]	0.647
Blood loss, mL	1690 [1560–3640]	1834 [1275–2575]	0.336
Norepinephrine administered, (μg/kg/min)/n	0.08 [0.03–0.10]	0.04 [0.02–0.09]	0.104
Vasopressin administered, (IU)/n	0 [0–20.0]	0 [0–0]	0.122
Milrinone administered, (mg)/n	47.4 [0–67.2]	48.0 [0–76.8]	0.401

Data are expressed as mean ± SD, median [IQR], or number of patients. RBC = red blood cell; FFP = fresh frozen plasma; NEPI = norepinephrine; ECMO = extracorporeal membrane oxygenation; OR = operating room.

**Table 3:** Perioperative renal function and post-operative 30-day morbidity.

Variables	Sevoflurane Group (n = 29)	Propofol Group (n = 30)	*p* Value
Baseline serum creatinine, mg/dL	0.64 ± 0.26	0.60 ± 0.16	0.497
AKI stratified by AKIN criteria	11 (38)	4 (13)	0.030
Stage I	7 (24)	4 (13)	0.287
Stage II	3 (10)	0	0.112
Stage III	1 (3)	0	0.492
Serum NGAL, ng/mL			0.039 ^a^
Baseline	115.3 ± 88.0	118.5 ± 94.2	0.938
Immediately after surgery	184.4 ± 141.6	103.3 ± 74.6	0.012
After 24 h	193.2 ± 168.1	108.7 ± 98.7	0.037
After 48 h	98.8 ± 0.5	84.5 ± 65.4	0.047
Serum Cystatin C, mg/L			0.223 ^a^
Baseline	0.93 ± 0.48	0.74 ± 0.41	0.110
Immediately after surgery	0.77 ± 0.43	0.67 ± 0.32	0.284
After 24 h	1.07 ± 0.58	0.88 ± 0.36	0.134
After 48 h	1.34 ± 0.72	1.00 ± 0.35	0.084
Furosemide usage within 48 h, mg	120 [60–185]	100 [40–160]	0.132
RRT within 48 h	3 (10)	0	0.237
RRT during hospitalization	6 (21)	1 (3)	0.052
In-hospital morbidity			
Reoperation for bleeding	3 (10)	2 (7)	0.671
Rejection	2 (7)	2 (7)	1.000
Stroke or transient ischemic attack	2 (7)	1 (3)	0.612
Primary graft dysfunction grade 3	14 (48)	8 (27)	0.086
Duration of prolonged ECMO, h	55 [35–98]	36 [31–85]	0.329
Duration of mechanical ventilation, h	156 [96–372]	132 [94–288]	0.533
ECMO re-insertion	4 (14)	1 (3)	0.195
Length of ICU stay, day	8 [7–14]	10 [7–17]	0.490
Length of hospital stay, day	36 [22–64]	29 [23–46]	0.255
Mortality	2 (7)	0	0.492

Data are expressed as mean ± SD, median [IQR], or number of patients. AKI = acute kidney injury; AKIN = acute kidney injury network; ECMO = extracorporeal membrane oxygenation; ICU = intensive care unit; NGAL = neutrophil gelatinase-associated lipocalin; RRT = renal replacement therapy. ^a^ P_GroupxTime_ = *p*-value of the group and time interaction obtained by the linear mixed model.

## Data Availability

Not applicable.
